# Intrinsic molecular insights to enhancement of biogas production from kitchen refuse using alkaline-microwave pretreatment

**DOI:** 10.1038/s41598-019-42471-9

**Published:** 2019-04-12

**Authors:** Puneet Kumar Singh, Suresh K. Verma, Sanjay Kumar Ojha, Pritam Kumar Panda, Haragobinda Srichandan, Ealisha Jha, Snehasish Mishra

**Affiliations:** 1 Bioenergy Lab, School of Biotechnology, KIIT University, Bhubaneswar, 751024 India; 2 Department of Biophysics, AIIMS, New Delhi, India; 3 Division of Pediatric Hematology and Oncology, University Children’s Hospital, University of Freiburg, Freiburg, 79106 Germany; 4 Memorial University of Newfoundland, Department of Physics and Physical Oceanography, St. John’s, Newfoundland and Labrador, NL A1C 5S7 Canada

## Abstract

The current study analyzed and optimized the concentration of NaOH for alkaline pretreatment of kitchen refuse for biogas production. Also, the benefits of microwave assistance in enhanced biogasification of kitchen refuse were evaluated. The TS, VS and structural changes were compared using standard experimental techniques. Molecular dynamics was investigated for the molecular level changes leading to higher biogasification in NaOHmicrowave combined pretreatment. The methane and biogas yields were calculated to validate the benefits of microwave assistance in efficient biogasification. The NaOH-microwave combined pretreatment showed higher VS production. Microwave treatment degraded and removed lignin more efficiently. Molecular dynamics studies revealed the induction of configurational instability in lignin and cellulose molecules with variable temperatures. The methane and biogas production increased with 6% NaOH concentration, and decreased at higher NaOH concentration till 10%. Microwave assistance declined the required NaOH concentration further to 4%. Thus, as compared to 6% NaOH concentration required for an efficient pretreatment, the kitchen refuse was efficiently pretreated with 4% NaOH concentration when combined with a 30 min duration microwaving. The experimental and computational data provided a detailed analysis proposing an optimized, novel and promising method to pretreat kitchen refuse for efficient and enhanced biogas production.

## Introduction

The demand for environmental safety and chemosphere protection because of harmful effects by the use of non-renewable sources of energy has risen in leaps and bounds in recent days. Increasing energy demand and global warming has reinforced to develop alternative and renewable energy resources^1,2^. Use of renewable energy includes the use of eco-friendly raw materials derived from the ecosystem and their waste^3^. Among different sources, Biogas has been recognized as a most promising and reliable source of energy^4^. The benefits of using biogas include economic feasibility having the lowest financial input of output energy, eco-friendly in nature and unlimited in potential^5^. Moreover, total organic waste is utilized without any negative impact^6^. The use of biogas has solved the problem of energy resource in a ‘zero-waste’ manner via generation of energy and biomanure.

The production of biogas is implicated by the action of consortia of various groups of anaerobic bacteria to the organic wastes produced by different means. Hence, one most important benefit of biogas production is the utilization of organic wastes being produced by anthropogenic activities. The basic constituent of biogas includes combustible hydrocarbons that can produce heat and energy on burning. A major factor that influences the productivity of biogas is the feedstock. Different types of feedstock that has been utilized are basically cattle dung, human wastes, organic fraction of municipal solid wastes, agricultural wastes and poultry litters^7^. The feed stocks are being chosen according to the productivity.

In last few decades, apart from the utilization of this feedstock, kitchen refuse (KR) have emerged as one of the potential feedstock for biogas production^8^. KR are defined as organic wastes with food scraps and wastes produced by day to day life food consumption and cooking wastes^9,10^. They contain a variety of organic sources such as polymers, carbohydrates, proteins and many others. These sources are the inspiration for utilizing them for biogas production via anaerobic digestion. The recycling rate of food wastes are very low (~13%), municipal solid waste content largest amount of it after other recyclable waste. It is the major source of foul smell and ground water contamination because of its organic matter and moisture content^11^. Previous reports have shown the potential production of 367 m^3^ of biogas per dry ton (about 65% methane and energy content of 6.25 KRh/m^3^ of biogas annually)^12,13^.

The anaerobic digestion includes basic processes like hydrolysis, acidogenesis, acetogenesis, and methanogenesis. Among these different steps, the most critical and rate-limiting one is the hydrolysis^12^. Hydrolysis refers to a process of breakdown of complex molecules of organic wastes like KR into monomers which require a longer time for solubilization. The process needs assistant to reduce the duration of hydrolysis which is commonly termed as ‘pretreatment’. The pretreated organic substrate has been reported to exhibit enhanced soluble oxygen demand (SCOD) which is an important parameter in the determination of productivity. It was reported that 6 hours chemical pretreatment of 62.0 mEq/L was found to be optimized with 11.5% increase in SCOD and 150 mL CH_4_/g VS (172% higher) as compared to an untreated substrate^14,15^. Considering the fact, pretreatment of KR has been brought into the process by different researchers through different techniques for the higher production of biogas yield^16^. Being rich in organic content it could serve as an ideal biogasification candidate^17^. Alkaline (NaOH) pretreatment has shown better results in the solubilization of complex organic matter^18^.

The pretreatment processes can be classified as thermal, mechanical, chemical and biological pretreatments according to the use of different natural factors. Majority of the findings associated with pretreatment of organic wastes were belonging to mechanical, thermal and chemical methods accounting for 33, 24 and 21%, respectively^19^. Microwave irradiation causes fast heating of substrate which results in rapid solubilization which further helps in enhancing the SCOD. This increased amount of SCOD helps in expediting anaerobic digestion^20^. The addition of alkali to the substrate before microwave irradiation helps in softening of the cell wall and releasing the soluble part of the cells, thereby facilitating disruption^21^.

Researchers are on regular quest to develop new methods for pretreatment of KR for enhanced biogas production. However, the need for higher efficiency is still not achievable. Moreover, a molecular level analysis is lacking in earlier reports which are a crucial point in order to analyze for the efficacy and efficiency of pretreatment. In view of these, this work describes a novel method of pretreatment of KR using alkaline-microwave pretreatment in order to achieve higher biogas production and analyze the process for the first time at the molecular level through *in silico* approaches. The degradation of molecular biomolecules like cellulose and lignin were evaluated with daily cumulative biogas production analysis to verify and validate the fact.

## Material and Methods

### Sample collection and preparation

KR was collected from the hostel canteen of the School of Biotechnology, Kalinga Institute of Industrial Technology (KIIT), Bhubaneswar, Odisha, India. The sample was brought to the laboratory and stored in polythene bags at 4 °C after blending in a kitchen blender. Major fractions of the KR were peelings, refuse of vegetables and cooked rice. The inoculum for the study was collected from a running KR-based biogas plant at the Kalinga Institute of Social Sciences (KISS). The total solids (TS) content of the seed inoculum was 2%. All the data generated or analyzed during this study are included here.

### Pretreatment

Pretreatment of KR was performed with different concentrations (0–10%) of NaOH for 24 h at room temperature in one set and microwave oven exposure of 30 min with the similar concentrations of NaOH submerged KR in another set to check the additive effect of microwave pretreatment to NaOH for enhanced biogas production in the pretreated KR^22^.

### NaOH pretreatment at room temperature

Finely chopped 100 g KR was submerged in 300 mL NaOH solution (0, 2, 4, 6, 8 and 10%) at 25 °C for 24 h in 500 mL reagent bottles. After 24 h time period, NaOH was filtered using a vacuum filter. The samples were then washed with tap water to pH 7.0 and dried in hot air oven at 105 °C for 4 h. After the sample dried, it was packed in polythene bags and kept at room temperature until further study^22^.

### NaOH-microwave pretreatment

100 g of KR was suspended in 300 mL NaOH solution of different concentrations (0, 2, 4, 6, 8 and 10%). The reagent bottles with KR Suspended in NaOH were kept on the glass plate of a microwave oven (LG Electronics India Pvt. Ltd.) and irradiated (720 W, 180 °C) for 30 min^16^ and NaOH solution was filtered using a vacuum filter. After filtration, the samples were washed with tap water to pH 7.0 and dried in hot air oven at 105 °C for 4 h. After the sample dried, it was packed in polythene bags and kept at room temperature till further study.

### Analytical methods

The TS, volatile solids (VS), moisture and ash contents were analyzed following standard procedures^20^. TS and moisture content of the samples were analyzed by placing 1 g of the sample in a hot air oven (Bio Techno Lab, India) at 105 °C for 1 h followed by cooling in a desiccator. The procedure was repeated until a constant weight was achieved. The VS and ash contents of KR were determined by placing 1 g of the sample in borosilicate crucibles in a muffle furnace at 550 °C for 30 min.

The volume of the biogas formed in the digesters was collected through a simple downward water displacement technique wherein the collected gas amount (mL) was measured based on the amount of water displaced from the gas collection unit. The biogas was qualitatively analyzed using an online push-fill type of sample injector gas chromatograph (Nucon-5700) to quantify the constituent gases, such as methane, carbon dioxide, and hydrogen sulfide. Porapak QS column (length 2.0 m; #80–100) was used with a TCD detector at 200 mA. The oven, injector and the detector temperature were maintained at 40, 90, and 120 °C, respectively. Hydrogen, the carrier gas, had a flow rate of 35 mL/min. The daily cumulative methane contents and the corresponding theoretical value of the methane content (mL/g VS) were worked out based on the mean average values of the percent methane content as per the chromatograph peaks.

### Experimental set-up

Biogas production test of KR was performed in 1L glass digesters at 40 °C for a 30-day period. 50 g of KR samples of each pretreatment (NaOH at room temperature and NaOH-microwave assisted) were placed in digesters in triplicate along with 50 mL of filtered slurry from running biogas plant based on KR and 100 mL of tap water. Digester based on the untreated sample was kept as a control to study the effect of pretreatment on biogasification of KR with the same volume of water and slurry. The headspace of the digesters was flushed with nitrogen gas to ensure the anaerobic environment inside it and capped with rubber sealing. The volume of biogas measured by downward water displacement method in a vessel and the opening of the vessel is connected with the online-GC system to analyze the quality of biogas. The experimental setup was designed as earlier reported^22^, with some modification.

### Analysis of buffering capacity

The buffering capacity of the substrate resembles its resistance to pH change. 1 g of untreated and treated samples of KR was taken and submerged into 80 mL of distilled water for 2 h. Then the initial pH of each sample was measured and maintained to 7.0 with 0.1 N NaOH when found lower than 7.0. The volume (mL) used to maintain the pH to 7.0 was referred to as titratable alkalinity. Then, the pH of each sample mixtures was changed to 4.0 from 7.0 with the help of 0.1 N HCl, and the volume (mL) required for this, was referred as titratable acidity^23^. The acid and base buffering capacities of each sample were calculated with the help of the following equations:

1 $$Acid\,buffering\,capacity\,(mL)=\frac{{\rm{Titratable}}\,{\rm{acidity}}}{7-4}$$

2 $$Base\,buffering\,capacity\,(mL)=\frac{{\rm{Titratable}}\,{\rm{alkalinity}}}{7}$$

### Water swelling capacity

Water swelling capacity estimation of untreated and treated KR was performed by using 0.1 g of sample kept in 10 mL of water for 1 h. The sample was filtered after 1 h time and the weight difference were calculated as per equation,

3 $$Water\,swelling\,capacity\,(g/g)=\,({{\rm{W}}}_{2}-{{\rm{W}}}_{1})/{{\rm{W}}}_{1}$$

where, W_1_ is the weight of the dry sample and W_2_ is the weight of the sample after swelling.

### FTIR analysis

**Figure 1:**
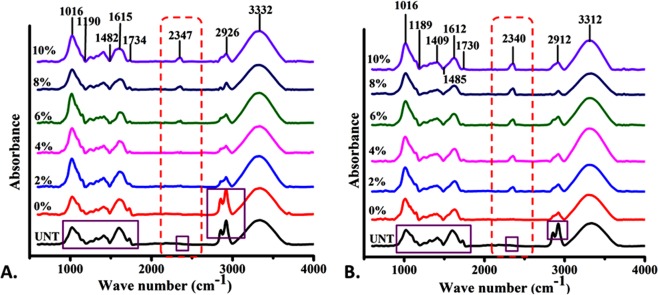
FTIR spectrum of KR substrate after pretreatment: (**A**) NaOH pretreatment of KR for 24 h; (**B**) microwave assisted NaOH pretreatment of KR for 30 min.

Analysis of functional groups present in untreated and treated KR was performed using FTIR (PerkinElmer Spectrum, Ver. 10.4.3, USA). The spectra were recorded within the range of 400–4000 cm^−1^ with the resolution of 4 cm^−1^, and the data are presented in Fig. 1.

### Scanning electron microscopy (SEM) of KR

The structural changes in KR before and after pretreatment was analyzed by SEM (Hitachi, Japan). The samples were mounted on carbon tape followed by gold sputtering for proper visualization. During analysis accelerating voltage, emission current, working distance, and vacuum were maintained at 15000 V, ~90000 mA, 14.0–14.7 mm, and 15 kV, respectively.

### Fiber analysis of KR

Cellulose, hemicellulose and lignin determination of KR was performed as per the standard method^24^. 1 g finely powdered sample was taken in 100 mL neutral detergent solution for estimation of neutral detergent fiber (NDF). 0.5 g of sodium sulfite was added to it at room temperature and the solution was refluxed in reflux condenser for 60 min from the onset of boiling. The solution was filtered and washed with warm distilled water followed by washing with cold acetone. The crucible was dried in a hot air oven for 1 h at 105 °C. The dried crucible with the sample was placed in a muffle furnace and ignites at 550 °C for 30 min. and NDF (%) calculation was done as per the equation stated below:

4 $$NDF( \% )=\frac{(({\rm{Wt}}.\,{\rm{of}}\,{\rm{crucible}}+{\rm{NDF}})\mbox{--}{\rm{Wt}}.\,{\rm{of}}\,{\rm{crucible}})}{({\rm{Wt}}.\,{\rm{of}}\,{\rm{sample}})}\times 100$$

The acid detergent fiber (ADF) of KR was estimated by placing 1 g of the sample in crucible followed by addition of 100 mL ADF solution. The reflux of solution performed in the condenser for 60 min after the onset of boiling and the rest of the procedure is same as described in NDF estimation. Weight loss calculation was performed as follows:

5 $$ADF( \% )=\frac{(({\rm{Wt}}.\,{\rm{of}}\,{\rm{crucible}}+{\rm{ADF}})\mbox{--}{\rm{Wt}}.\,{\rm{of}}\,{\rm{crucible}})}{({\rm{Wt}}.\,{\rm{of}}\,{\rm{sample}})}\times 100$$

The acid detergent lignin (ADL) estimation of KR was performed with the remaining residue of ADF by addition of 72% H_2_SO_4_ in the same crucible and mix it well. The resulting solution was filtered and obtained residue washed twice with hot water. The residue was then dried in a hot air oven at 105 °C for 3 h followed by igniting at 550 °C in muffle furnace for 10 min. Cellulose, hemicellulose, and lignin were calculated as per the following equations:

6 $$Hemicellulose\,( \% )={\rm{NDF}}\, \% -{\rm{ADF}}\, \% $$

7 $$Cellulose\,( \% )=\,\frac{(({\rm{Wt}}.\,{\rm{of}}\,{\rm{crucible}}+{\rm{ADF}})\mbox{--}{\rm{Wt}}.\,{\rm{of}}\,{\rm{crucible}}+{\rm{Lignin}})}{({\rm{Wt}}.\,{\rm{of}}\,{\rm{sample}})}\times 100$$

8 $$Lignin\,( \% )=\frac{(({\rm{Wt}}.\,{\rm{of}}\,{\rm{crucible}}+{\rm{Lignin}})\mbox{--}{\rm{Wt}}.\,{\rm{of}}\,{\rm{crucible}}+{\rm{Ash}})}{({\rm{Wt}}.\,{\rm{of}}\,{\rm{sample}})}\times 100$$

### Auto-fluorescence imaging

To understand the structural changes occurred before and after pretreatment, auto-fluorescence imaging of KR was performed using inverted-fluorescent microscope (Olympus, Japan). The intensity of the red and green fluorescence was calculated using image J software.

### *In silico* analysis

Lignin (Organosolv) bearing PubChem CID 73555271 and Cellulose (PubChem CID:16211032) has been downloaded and subjected for temperature-dependent molecular dynamics simulation. Initially, the chemical structure has been geometrically optimized using Argus Lab to attain minimum potential and lowest free energy by geometry convergence function. The calculations were based on Hartree-Fock (HF calculation method)^25^. After the initial steps of geometry optimization, the minimized structures were administered with ZINDO^26^ (Zerner’s Intermediate Neglect of Differential Overlap) CI (Configuration Interaction) method to calculate the UV-Vis electronic absorption spectra of the molecules. These methods are used to calculate the ground state properties such as bond lengths, bond angles and molecular orbitals for calculating excited states. CI calculations referred to the post-Hartree-Fock linear variation method imposing a reference state of the molecule plus a small set of single electron excitations within a selected active space.

For temperature-dependent molecular dynamics simulation, the optimized chemical structures were subjected to Dreiding force field with integrator step Velocity Verlet using Marvin Sketch. The structures were simulated at 0 K, 30 K, 373.15 K, 400 K, 500 K, 600 K, 700 K to calculate the lowest potential energy (minimum free energy) attained during simulations. The basis of these empirical/quantum methods imposed on these structures was to evaluate the states of configuration attained during temperature variations.

## Results and Discussion

### Assessments of solids and moisture content

In order to assess the changes in total biomass content of KR after NaOH, and NaOH followed by microwave pretreatment. Different types of solid content (total solid and volatile solid) along with moisture content were assessed. As detailed in Supplementary Tables S1 and S2, the total solid content (TS) exhibited a decreasing trend as the NaOH concentration increased. However, the reduction was higher when this chemical pretreatment was followed with the microwave treatment. It was 25 ± 0.1, 24.66 ± 0.20, 23.76 ± 0.98, 23.33 ± 0.05, 23.23 ± 0.15 unit on 0, 2, 4, 6, 8 and 10% microwave + NaOH pretreatment respectively as compared to 25.10 ± 0.20, 25.10 ± 0.1, 24.7 ± 0.1, 24.5 ± 0.2, 24.43 ± 0.05 and 24.2 ± 0.1 unit in NaOH pretreatment alone. While analyzing the volatile solids (VS) contents in 0, 2, 4, 6, 8 and 10% NaOH pretreatments, the VS percentage significantly increased from 16.03 ± 0.20 to 16.53 ± 0.15, 17.03 ± 0.20, 18.3 ± 0.20, 18.53 ± 0.15, 18.73 ± 0.15 units. Interestingly, the percent VS content increased by 17.13 ± 0.2, 18.36 ± 0.15, 20 ± 0.52, 20.06 ± 0.11, 20 ± 0.36, 20.13 ± 0.35 units when microwaving followed NaOH treatment. As a result, the VS/TS ratio was found to be favorably enhanced on a combined microwave-alkali pretreatment as compared to the alkali-only pretreatment (Supplementary Tables S1 and S2). On a similar trend, the ash content was significantly reduced on microwave-alkali pretreatment compared to the alkali-only pretreatment. The percent moisture content increased by 75 ± 0.1, 75.33 ± 0.20, 76.23 ± 0.98, 76.66 ± 0.05, 76.8 ± 0.1, 76.76 ± 0.15 units on microwave-alkali pretreatment as compared to 75.23 ± 0.75, 75.56 ± 1.06, 75.3 ± 0.1, 75.5 ± 0.2, 75.56 ± 0.57, 75.8 ± 0.1 units at 0%, 2%, 4%, 6%, 8% and 10% NaOH-only pretreatment, respectively. These data indicate towards an enhanced hydrolysis on microwave pretreatment. Similar observations on microwave pretreatment of swine manure have been reported earlier^22,27^. With reference to earlier reports and the obtained results, the significant changes in TS and VS along with moisture can be attributed to the breakdown of intermolecular bonding of the biomolecules like lignin, cellulose, hemicellulose and polymeric substances due to heat exhibited by microwave assisted with alkaline pretreatment^28^. However, the alkali pretreatment can be reasoned to the solubilization of particulate organic matter^29^. An increase in the moisture content indicated towards an increased hydrolysis and gradual complete breakdown of the biomolecules that expectedly assisted in the higher biogas production. Moreover, the data indicated towards a significant change in the buffering and swelling capacity of the KR substrate which was further verified through various experimental analyses.

### Assessments of change in the buffering and swelling capacity

**Figure 2:**
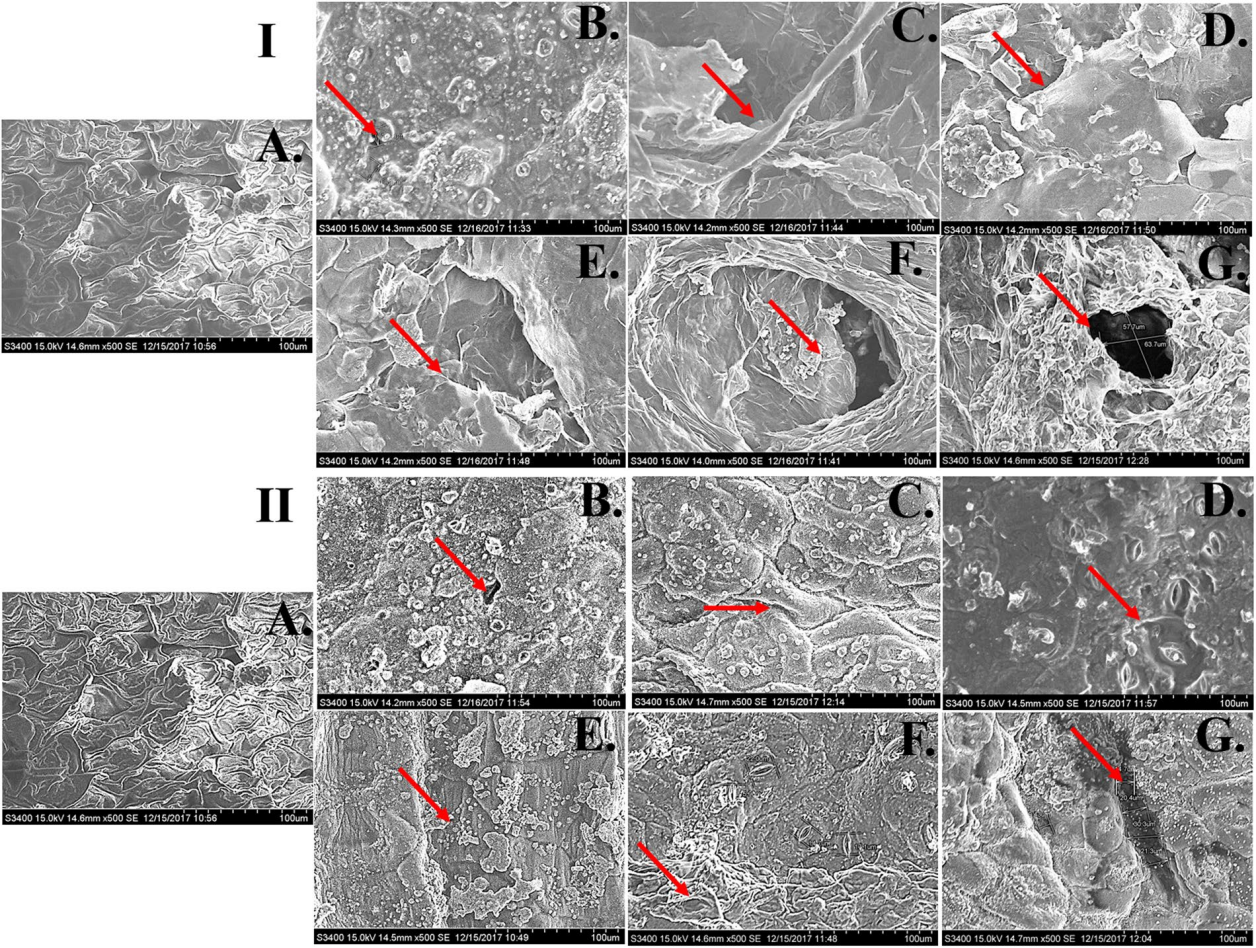
Scanning electron micrographs of pretreated KR: I. NaOH pretreated KR for 24 h at room temperature; II. Microwave-alkali pretreated KR for 30 min. (**A**) Untreated (**B**) 0% NaOH (**C**) 2% NaOH (**D**) 4% NaOH (**E**) 6% NaOH (**F**) 8% NaOH (**G**) 10% NaOH.

Buffering capacity is referred to the tendency of the substrate to resist the pH change which plays a crucial role in biogasification^30^. The capacities are generally measured as acid buffering and base buffering capacities, according to the ionic surroundings. These parameters were measured in KR pretreated with alkali and microwave assisted alkali pretreatments in order to assess their efficacy. As shown in Tables 1 and 2, the acid buffering capacity was found to be enhanced with percent increase in NaOH while it was significantly higher in KR pretreated with microwave-alkali pretreatment. In NaOH pretreated KR, the acid buffering capacity enhanced to 0.362, 0.504, 0.633, 0.726, 0.755 and 0.78 mL in 0, 2, 4, 6, 8 and 10% NaOH pretreatments respectively, while it was 0.404, 0.565, 0.724, 0.75, 0.773, 0.722 mL in microwave-alkali pretreatment. Interestingly, the base buffering capacities reduced in the latter process as compared to the former one. The data obtained during analysis indicated towards the neutralizing capacities of KR directing towards the change in its water swelling capacity. The low buffering capacity of feedstock resulted in the accumulation of fatty acids, and furthermore, led to a pH drop during biogasification^31^. Water swelling capacity was further assessed in KR pretreated with both processes to validate the assumption. The analysis revealed higher swelling capacity in case of microwave-alkali pretreatment as compared to alkali-only pretreatment (Tables 1 and 2). The water swelling capacity of pretreated substrate increased the pore volume of the surface that could hold more water compared to the untreated substrate, resulting in an increased accessibility of the hydrolytic enzyme/microbes, which could be reason for enhanced biomethanation in the pretreated KR^32,33^. The same logical view is supported through the substrate disruption patterns as observed in the electron photomicrograph (Fig. 2). The obtained results can be reasoned for higher efficacy of KR substrate for biogas production in microwave assisted alkali pretreatment process as compared to the only alkali pretreatment process.

**Table 1:** Acid buffering, base buffering and swelling capacities of NaOH pretreated KR.

Pretreatment (%)	Acid buffering capacity (mL)	Base buffering capacity (mL)	Swelling capacity (g/g)
UNT	0.338	0.042	1.38 ± 0.004
0	0.362	0.04	1.4 ± 0.004
2	0.504	NA	1.68 ± 0.016
4	0.633	NA	1.92 ± 0.024
6	0.726	NA	1.99 ± 0.012
8	0.755	NA	1.73 ± 0.012
10	0.78	NA	1.5 ± 0.009

UNT = Untreated, n = 3, ± SD, NA = Not applied.

**Table 2:** Acid-base buffering and swelling capacities of 30 min microwave-alkali pretreated KR.

Pretreatment (%)	Acid buffering capacity (mL)	Base buffering capacity (mL)	Swelling capacity (g/g)
UNT	0.338	0.042	1.38 ± 0.004
0	0.404	0.034	1.61 ± 0.012
2	0.565	NA	1.92 ± 0.020
4	0.724	NA	2.16 ± 0.012
6	0.75	NA	2.03 ± 0.016
8	0.773	NA	1.93 ± 0.012
10	0.722	NA	1.81 ± 0.012

UNT = Untreated, n = 3, ± SD, NA = Not applied.

### Analysis of structural modification in KR

To analyze and compare the structural modification in KR after pretreatments, the samples were subjected to different standard techniques like FTIR and SEM. As shown in Fig. 1 and Supplementary Table S3, a gradual change in transmittance peak was observed in FTIR spectrum of the substrate treated with alkali-only and microwave-alkali pretreatments. Significant peaks were found at 1016, 1189, 1409, 1612, 2340, 2912 and 3312 cm^−1^ corresponding to C-H, C-O-C and C = O bond stretching. The peaks were found to be shifting indicating towards a higher stretching of bonds, revealing structural modification in the biomolecules like lignin, hemicellulose and cellulose. The deviations of the peak were higher in microwave-alkali as compared to the alkali-only treated KR. On a significant note, the peak present at 2340 cm^−1^ corresponding to C-H stretching in cellulose was higher (Fig. 1). Further, the structural modification analysis performed with the help of scanning electron microscopy (Fig. 2) revealed well arranged and smooth surface in the untreated sample (Fig. 2I,II) whereas the surface of the pretreated samples was rough, disorganized and seemingly disintegrated (Fig. 2IB–G), while comparing the structural organization of the untreated and alkali-treated ones. The disruption (marked by red arrow) and the disorganization increased as the percentage NaOH concentration increased. Disruption of the outer layer near the stomatal region was also observed. A clear distinctive higher disintegration was observed (Fig. 2IIB–G) in microwave-alkali pretreated KR as compared to the alkali-only counterpart. The disintegration and an increase in the porosity of the sample is attributed to the breakdown of the surface cellulose, hemicellulose and other fibrous structure. This breakdown would further increase the efficiency of biogas production due to an enhanced enzymatic/microbial action^34^. The increased cracks and big pores on the surface of KR due to microwave-alkali pretreatment can be reasoned to the breakdown of lignocellulose component at increased temperature (due to microwaving). Physical measures like mechanical ‘sonication’ and temperature reportedly cause such effect on the substrate^35^. With reference to the previous reports and the data obtained, it can be safely concluded that the a combined microwave-alkali pretreatment helps in cell wall and biomass degradation, leading to an enhanced biogasification.

### Qualitative analysis of KR by auto-fluorescence

**Figure 3:**
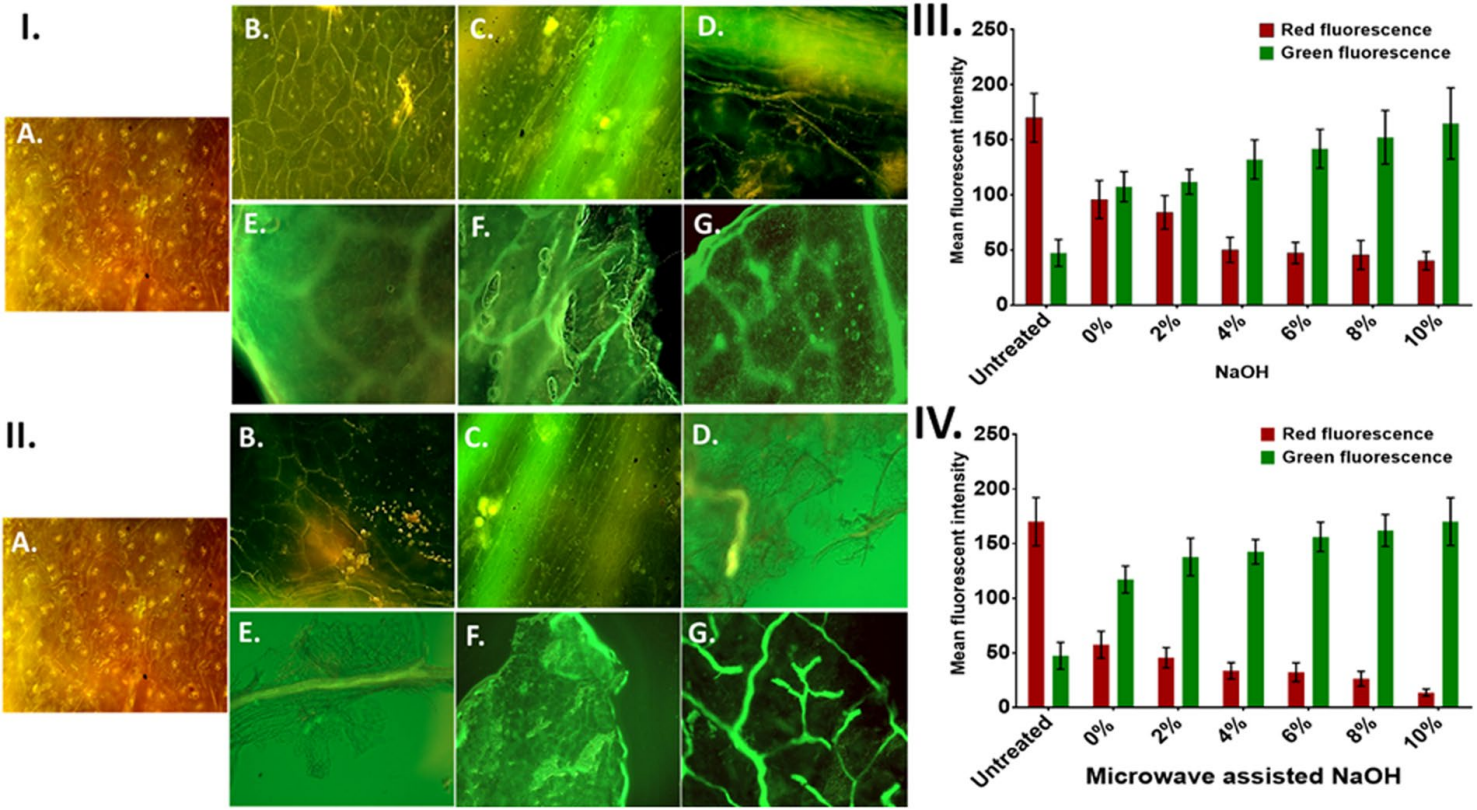
Auto fluorescence images of pretreated KR: I. NaOH pretreatment of KR for 24 h at room temperature; II. Microwave-alkali pretreated KR for 30 min. (**A**) Untreated (**B**) 0% NaOH (**C**) 2% NaOH (**D**) 4% NaOH (**E**) 6% NaOH (**F**) 8% NaOH (**G**) 10% NaOH. III. IV. The bar-graph alongside shows the change in the red and green fluorescence intensity depicting lignin and cellulose contents; this alteration in the intensity of red and green fluorescence was calculated using image J software.

Auto-fluorescence study was used to perform a qualitative analysis of pretreated KR substrate. The study was based on the exhibition of green fluorescence by the cellulose content. The exhibition of green fluorescence by plant biomass due to bound ferulic acid in cell walls and carbohydrates like cellulose is reported^36^. Cellulose reportedly shows fluorescence at 405 nm excitation. Hence, with reference to these reports, a change in the green fluorescence was attributed to the surface cellulose exposure. Lignin reportedly exhibits red fluorescence at 510–560 nm wavelength excitation^37^. Hence, the intensity of red fluorescence can be reasoned for the surface lignin exposure, degradation of which is a denotation of delignification. The untreated KR was found to exhibit higher red fluorescence (Fig. 3) indicating towards the presence of higher lignin contents (Figure 3IA, 3IIA). Figure 3IB–G,III showed a decline in red fluorescence and an increased green fluorescence in KR with alkali-only pretreatments at various concentrations. Similar trend was found in case of microwave-alkali pretreatment (Fig. 3IV). However, the increase in green fluorescence was higher as compared to alkali-only pretreatment. The observation can be attributed to the fact that NaOH pretreatment helped in concentration-dependent delignification which was enhanced when assisted with the microwave pretreatment. Increased green fluorescence can be reasoned to the exposure of cellulose at the surface, also attributable to delignification. The results were in line with the previous reported fluorescence intensity of microbial pretreatment^38^.

### Fibre analysis of KR

**Figure 4:**
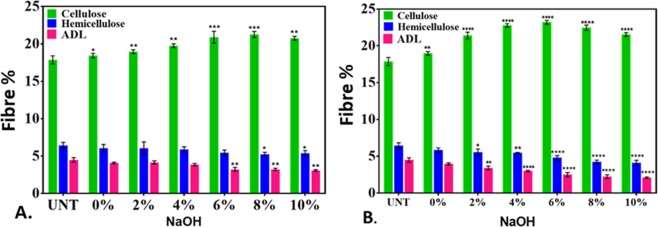
The cellulose, hemicellulose and lignin analyses of: (**A**) NaOH-pretreated KR for 24 h at room temperature; (**B**) Microwave-alkali pretreated KR for 30 min. The values represent mean ± SD of three independent experiments. Statistical analyses were performed using two-way ANOVA. *P < 0.01 denotes significant change from the untreated, obtained from ANOVA. Number of * presents the degree of significance.

Microscopic analysis indicated towards the qualitative change in the composition of fibres like cellulose, hemicellulose, and lignin in KR on alkali-only and microwave-alkali pretreatments. The observation was further validated by quantitative experimental analyses. As observed in Fig. 4A, the cellulose and hemicellulose contents in KR were found to be significantly unchanged in alkali-only pretreatment while the lignin (ADL) significantly reduced as the percent NaOH increased. Similar trends were observed in the microwave-alkali pretreatment. However, lignin reduction was higher in microwave-alkali pretreatment. As observed, the 5% lignin content in the untreated sample reduced to 4.5% in 10% alkali-only pretreatment, while it was up to 3% in its microwave-alkali pretreatment counterpart. The data reinforced the microscopic analysis, and were in line with earlier reports^21^.

**Figure 5:**
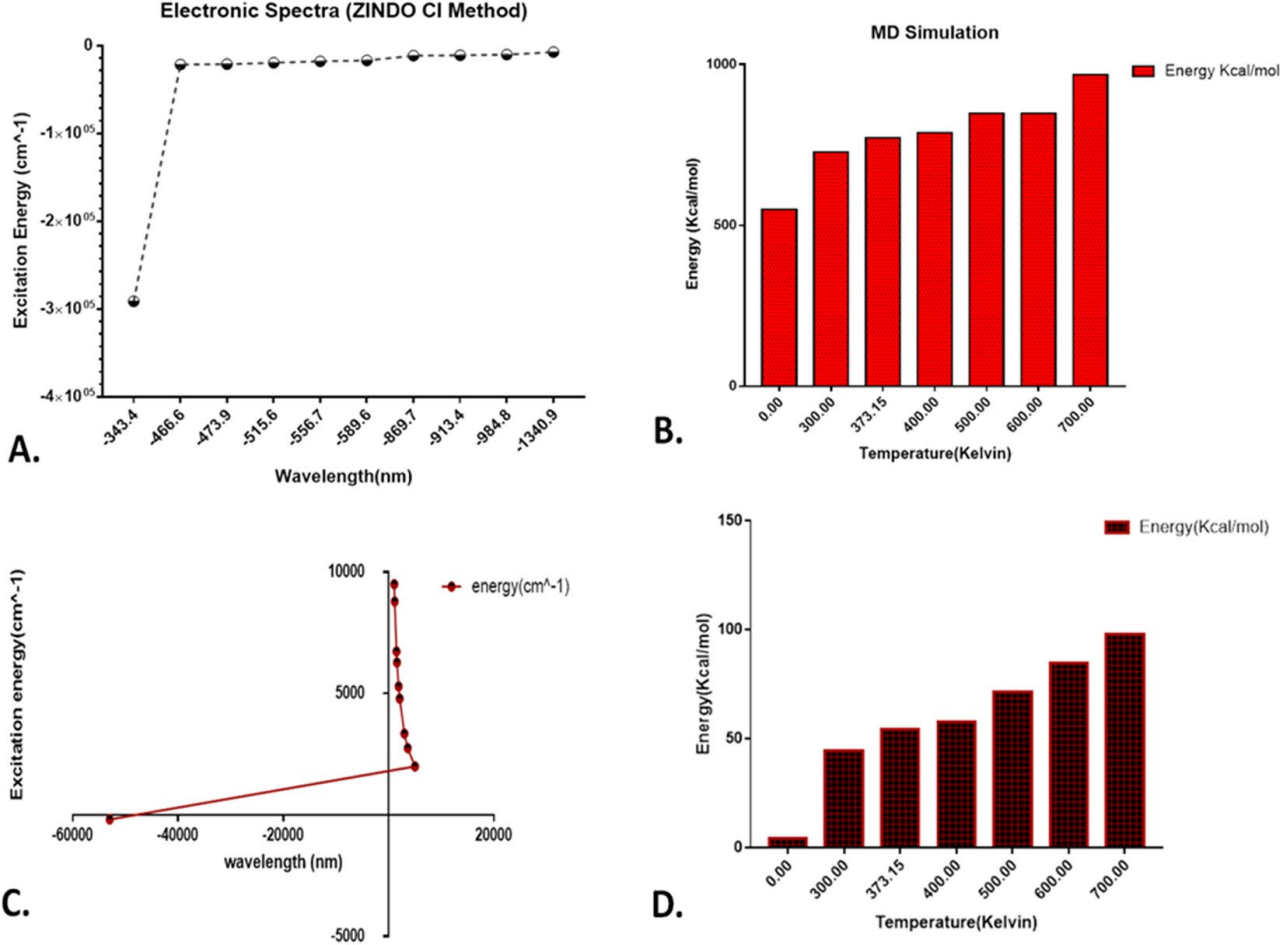
Computational analysis of lignin and cellulose: (**A**,**C**) Excitation energy of lignin and cellulose as determined by ZINDO CI method; (**B**,**D**) Molecular dynamics free energy of lignin and cellulose at different temperatures.

**Figure 6:**
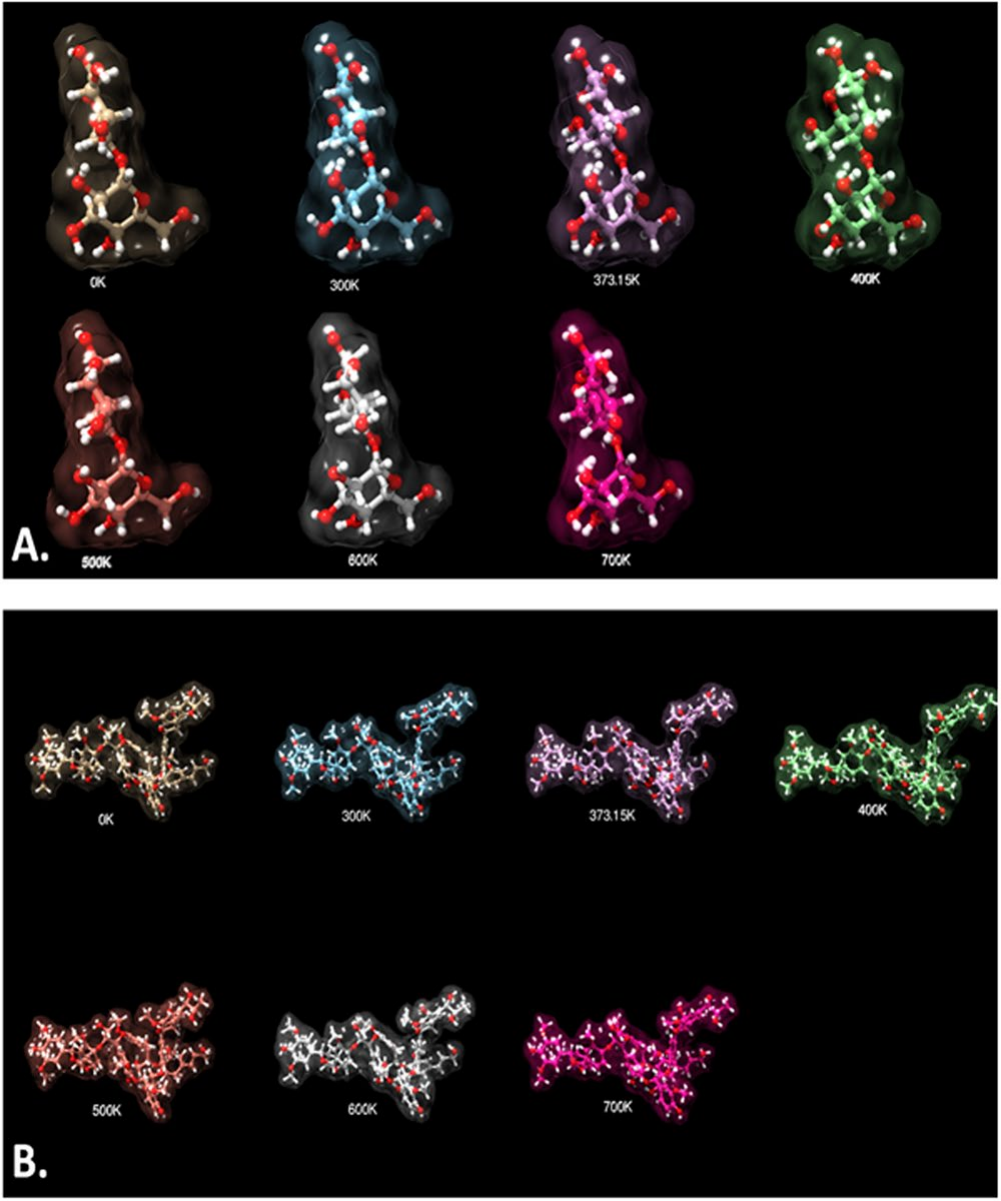
Configurational analysis of: (**A**) Lignin (**B**) cellulose at different microwave temperatures as determined through *in silico* molecular dynamics.

In order to understand the role of microwave assistance and evaluate the molecular dynamics of cellulose and lignin on microwave-alkali pretreatment, an *in silico* analysis was done through computational approach. As determined by the ZINCO-CI method, the electronic UV-Vis spectra of the lignin molecule were found to the geometrically stable with constant energy and bond length at untreated condition while it was found to be increasing with the wavelength in case of cellulose molecule (Fig. 5A,C). Free energy of the lignin molecule was found to increase with an increase in temperature as hypothesized during the microwave pretreatment (Fig. 5B). Similarly, the cellulose molecules were found to have an increasing free energy state with increased temperature (Fig. 5D). Figure 6 shows the real state dynamics of the configuration of lignin (Fig. 6A) and cellulose (Fig. 6B) molecule with an increase in temperature. It was clearly observed that the configuration of both the molecules was found to be disturbed with gradual increase in temperature. The data supported the results depicting the crucial role of microwave in delignification of KR. The exposed cellulose content can be reasoned for the enhancement in biogas production efficiency.

### Effects of pretreatment methods on the methane yield

**Figure 7:**
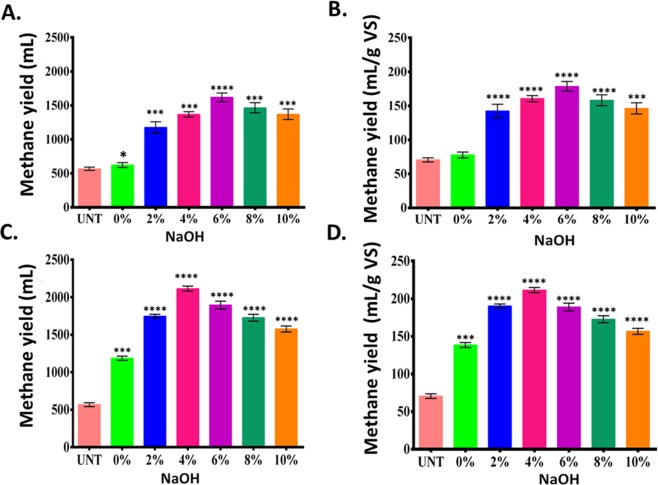
Methane yields in KR: (**A**) daily cumulative methane yield, and (**B**) methane (mL/g VS) production in NaOH-pretreated KR; (**C**) daily cumulative methane yield, and (**D**) methane (mL/g VS) production in microwave-NaOH pretreatment for 30 min. The values represent mean ± SD of three independent data. Statistical analyses were performed using two-way ANOVA. *P < 0.01 denotes significant change from the untreated, obtained from ANOVA. Number of *presents the degree of significance.

Evaluation of daily cumulative methane yield and methane yield per gram of VS was further performed in order to understand the efficacy of the pretreatment methods and the amount of daily cumulative methane and methane (mL/g VS) was calculated as per earlier report^39^. As shown in Fig. 7A, the daily cumulative methane yield (mL) increased from 540 mL in the untreated to 640, 1220, 1450, and 1640 mL with an increasing (2–6%) NaOH pretreatment. Interestingly, the cumulative yield again decreased to 1410 and 1320 mL when NaOH increased further to 8 and 10%, respectively. The methane yield in alkali-only pretreated KR resulted in 68, 80, 140, and 160 mL/g VS with 0, 2, 4, and 6% NaOH pretreatments respectively (Fig. 7B), while it further decreased to 154 and 146 mL as the NaOH percentage increased to 8 and 10%. Both the yields increased in the microwave-alkali pretreatments as compared to their alkali-only counterparts. Figure 7C,D show the cumulative and per gram VS methane yield of KR in microwave-alkali pretreatment. As observed, the cumulative methane yield initially increased to 1170, 1740 and 2130 in microwave-alkali pretreatment at 0, 2 and 4% NaOH which further decreased to 1890, 1750 and 1580 mL at 6, 8, and 10% NaOH. Methane yield (mL/g VS) also followed a similar trend, increasing from the initial 66 mL in the untreated substrate to 135, 192 and 210 mL in microwave-alkali pretreatment at 0, 2 and 4% NaOH. There was a further decline in microwave-alkali pretreatment at 6, 8 and 10% NaOH, to 192, 176 and 154 mL. The initial increase in alkali-only pretreatment at 6% NaOH is attributed to the contribution of –OH for hydrolysis of cellulose and lignin molecules, while a higher Na^+^ ion can be reasoned to inhibit the methanogenesis at higher NaOH concentration^40^. The availability of cellulose molecules in microwave-assisted pretreatment were higher, which must have been facilitated by up to 4% NaOH. A NaOH concentration above that must have inhibited the process due to the higher availability of Na^+^. Data indicated that a lower concentration of NaOH was optimal when assisted with microwave, and revealed the benefits of microwave-alkali pretreatment.

### Effects of pretreatments on the biogas yield

**Figure 8:**
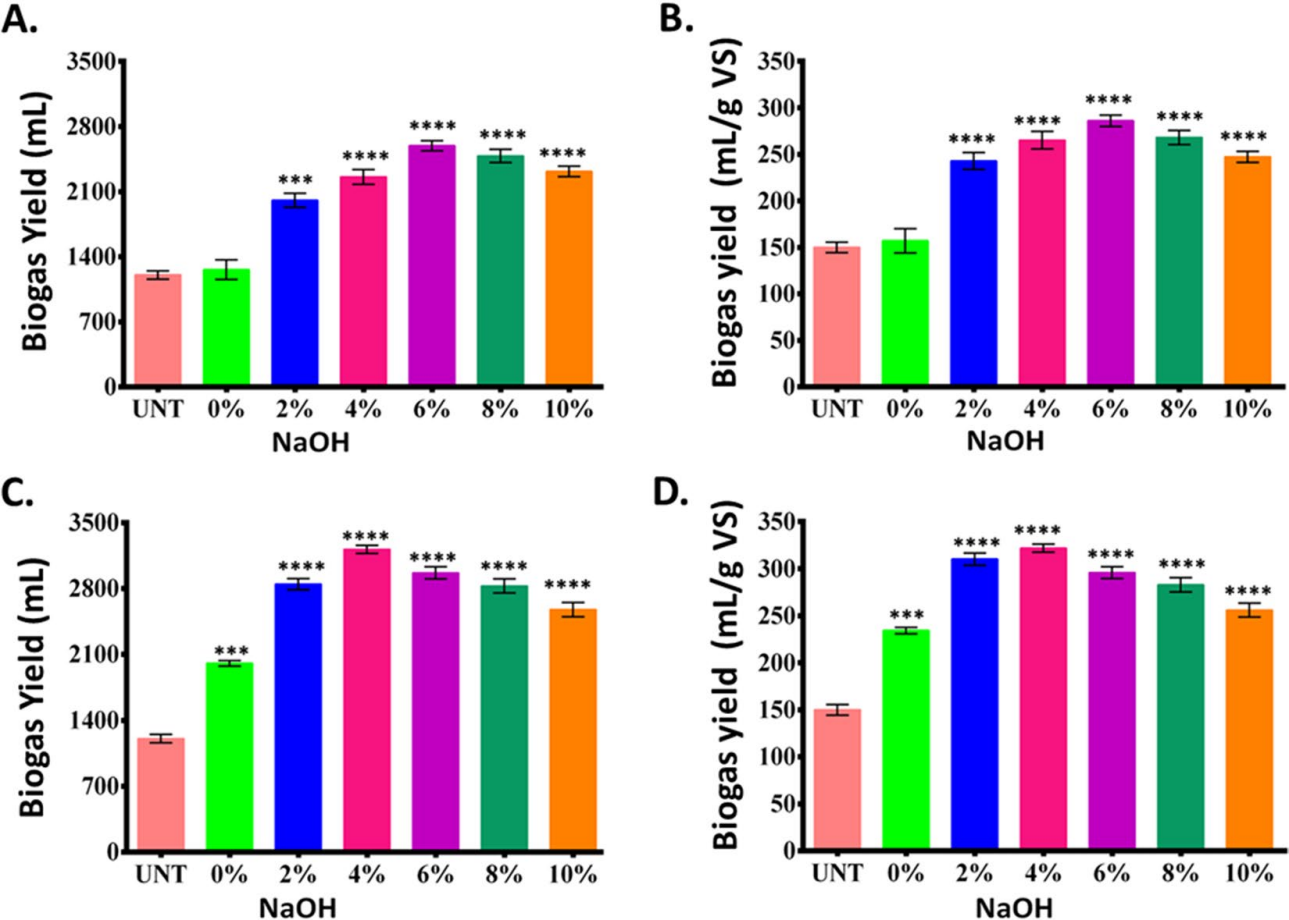
Biogas yields in KR: (**A**) daily cumulative biogas yield, and (**B**) biogas (mL/g VS) production in NaOH-pretreated KR; (**C**) daily cumulative biogas yield, and (**D**) biogas (mL/g VS) production in microwave-alkali pretreatment for 30 min. The values represent mean ± SD of three independent data. Statistical analyses were performed using two-way ANOVA. *P < 0.01 denotes significant change from the untreated, obtained from ANOVA. Number of * presents the degree of significance.

To explore the benefits of combined microwave-alkali pretreatment on the hydrolysis, the daily cumulative biogas and biogas (mL/g VS) production were investigated. The daily cumulative biogas production increased from 1190 mL in the untreated substrate to 1320, 2020, 2340 and 2560 mL in only-alkali pretreatment at 0, 2, 4, and 6% NaOH (Fig. 8), which declined to 2460 and 2300 mL at 8 and 10% NaOH. Evidently, similar trends were observed in case of per gram VS biogas yield which increased to 165, 240, 265 and 285 mL at 0, 2, 4 and 6% NaOH compared to the untreated’s 140 mL yield. Biogas yield increased to 1980, 2780, and 3210 mL in microwave-alkali pretreatment at 0, 2, and 4% NaOH. The yield also increased up to 230, 305 and 325 mL/g VS in microwave-alkali pretreatment at 0, 2, and 4% NaOH. However, a declination in the biogas yield was observed in both alkali-only and microwave-alkali pretreatments with a further increase of NaOH concentration up to 10%. The decline trend started even at 6% with the combined pretreatment as opposed to 8% in alkali-only pretreatment, both in biogas as well as methane yields. The universal observation of an initial increase and a later decrease in the biogas yield with the combined microwave-alkali pretreatment is attributable to the volatilization of cellulose, lignin, and hemicellulose^41^, as also observed in Fig. 4, and the decreasing trend in the TS contents. The presence of a higher percentage of Na^+^ can also be a reason for the decrease at higher NaOH concentration. Earlier report also states about a similar trend of biogas and methane production on alkali-only and microwave-alkali pretreatments^17^. Thus, it is concluded that microwaving increases the efficacy of NaOH pretreatment for biogas production and the related microbial processes due to assisted breakdown of polymers and generation of more volatile substance for biogas production. Thus, with various process optimizations, the technique could be a reliable intervention for increased biogas production at pilot-scale.

## Conclusion

The study revealed the benefit of using microwave-alkali pretreatment for enhanced biogasification of KR. Two different (alkali-only and microwave-alkali) pretreatments at different alkali concentrations on KR were compared. Different parameters were analyzed at substrate, molecular and final yield levels. As a universal observation, the TS value decreased while the VS level increased with ensue of biomethanation. Structurally, the KR was found to decompose and disintegrate at a higher level with microwave-alkali pretreatment. Molecular dynamic study revealed the role of temperature in the change in configuration of lignin and cellulose molecules owing to the pretreatment. The microwave-alkali pretreatment also beneficially affected the methane and biogas yields. The study, thus, provided a detailed insight into the optimum concentration of NaOH for pretreatment while emphasizing on the role of microwave in enhancing biogas yield.

### Ethical statement

This article does not contain any studies with human participants or animals performed by any of the authors.

## Supplementary information

Supplementary information

## Data Availability

The data and the protocols used in the manuscript are available with the corresponding author.
