# Supplementary material for: Intrinsic molecular insights to enhancement of biogas production from kitchen refuse using alkaline-microwave pretreatment
Source: Sci Rep. 2019 Apr 12;9:5968. doi: 10.1038/s41598-019-42471-9 (PMC6461693; doi:10.1038/s41598-019-42471-9)
Supplement: Supplementary file 1 — Supplementary information [file 41598_2019_42471_MOESM1_ESM.docx]

**Intrinsic molecular insights to enhancement of biogas production from kitchen refuse using alkaline-microwave pretreatment**

Puneet Kumar Singh^1^, Suresh K Verma^1^, Sanjay Kumar Ojha^2^, Pritam Kumar Panda^3^, Haragobinda Srichandan^1^, Ealisha Jha^4^, Snehasish Mishra^1*^

*Corresponding author: snehasish.mishra@gmail.com

**Supplementary Information**

Table S1. Effect of NaOH-assisted microwave pretreatment on KR composition

| Proximate (%) | UNT | NaOH (%) | | | | | |
| --- | --- | --- | --- | --- | --- | --- | --- |
|  |  | 0 | 2 | 4 | 6 | 8 | 10 |
| TS | 25.53±.35 | 25±0.1 | 24.66±0.20 | 23.76±0.98 | 23.33±0.05 | 23.23±0.15 | 23.23±0.15 |
| VS | 16.06±0.20 | 17.13±0.2 | 18.36±0.15 | 20±0.52 | 20.06±0.11 | 20±0.36 | 20.13±0.35 |
| Ash | 9.46±0.55 | 7.86±0.11 | 6.3±0.34 | 3.76±0.46 | 3.26±0.05 | 3.5±0.23 | 3.11±0.2 |
| Moisture | 74.46±0.35 | 75±0.1 | 75.33±0.20 | 76.23±0.98 | 76.66±0.05 | 76.8±0.1 | 76.76±0.15 |
| VS:TS | 62.93±1.65 | 68.53±0.57 | 74.46±1.19 | 83.39±1.36 | 85.96±0.23 | 86.07±1.06 | 86.64±0.94 |

UNT=Untreated, n=3, ± SD

Table S2. Effect of NaOH pretreatment at room temperature on KR composition

| Proximate (%) | UNT | NaOH (%) | | | | | |
| --- | --- | --- | --- | --- | --- | --- | --- |
|  |  | 0 | 2 | 4 | 6 | 8 | 10 |
| TS | 25.53±.35 | 25.10±0.20 | 25.10±0.1 | 24.7±0.1 | 24.5±0.2 | 24.43±0.05 | 24.2±0.1 |
| VS | 16.06±0.20 | 16.03±0.20 | 16.53±0.15 | 17.03±0.20 | 18.3±0.20 | 18.53±0.15 | 18.73±0.152 |
| Ash | 9.46±0.55 | 9.06±0.32 | 8.53±0.152 | 7.66±0.115 | 6.36±0.05 | 5.9±0.1 | 5.46±0.05 |
| Moisture | 74.46±0.35 | 75.23±0.75 | 75.56±1.06 | 75.3±0.1 | 75.5±0.2 | 75.56±0.57 | 75.8±0.1 |
| VS:TS | 62.93±1.65 | 63.87±1.08 | 66.12±0.35 | 68.95±0.58 | 74±0.316 | 75.84±0.46 | 77.40±0.32 |

UNT=Untreated, n=3, ± SD

Table S3. FTIR spectrum analysis of untreated and pretreated KR representing the wavenumber, functional group and assigned biopolymers

| **Wavenumber (cm^-1^)** | **Functional group** | **Biopolymer/Assignment** |
| --- | --- | --- |
| 3312 | O-H Stretching | Lignin |
| 2912 | C-H Stretching | Cellulose |
| 2340 | C-H Stretching | cellulose |
| 1730 | C=O Stretching of acetyl or carboxylic acid | Hemicellulose and lignin |
| 1612 | C=C Stretching of aromatic ring | Lignin |
| 1485 | Asymmetric bending in CH_3_ | Lignin |
| 1409 | C-H Deformation | Lignin |
| 1189 | C-O-C Symmetric stretching | Cellulose |
| 1016 | C-H Deformation | Lignin |
